# 

**Published:** 1905-12-09

**Authors:** 


**NOTICE TO CORRESPONDENTS.**

All MSS., Letters, Books for Review, and other matters intended for the Editor should be addressed The Editor, "The Hospital," The Hospital Buildings, 28 & 29 Southampton Street, Strand, W.C.

The Editor cannot undertake to return rejected MS., even when accompanied by stamped directed envelope.

**Notice to Advertisers and Subscribers.**

All Advertisements, Orders for Copies of The Hospital, and Business Communications should be addressed to **THE MANAGER (Not to the Editor),** The Hospital, 28 & 29 Southampton Street, Strand, London, W.C.

The Cost of Subscription, post free, is at follows :—Per week, 3½d.; per quarter, 3s. 3d.; per half-year, 6s. 6d.; per annum, 13s. Foreign and Colonial :—Per annum, 19s. 6d., payable, in all cases, in advance.

**NOTICE.—Vol. XXXVIII. of "The Hospital" April to September 1905), in handsome cloth binding, will shortly be on sale at the office of this Journal, price 10s. 6d. Cases for binding the Numbers contained in this Volume 2s. Index to Vol. XXXVIII., 3½d., post free.**

**The Monthly Part for December is now ready. Price 1s., by post, 1s. 3d.**

### Medical Appointments.

**Alfred Edwin Ash**, M.D.Vict., District Medical Officer by the Honiton (Devon) Board of Guardians. **H. S. Ballantyne**, M.B., M.S.Edin., Certifying Surgeon under the Factory and Workshop Act for the Dalkeith District of the county of Edinburgh. **John Henry Blakeney**, L.R.C.P.Lond., M.R.C.S., District Medical Officer by the Cheltenham Board of Guardians. **Octavius Augustus Glasier Collins**, B.A., B.C.Cantab., Medical Officer to the Bath Statutory Hospital for Infectious Diseases at Combe Down. **E. A. Elkington**, M.B.Lond., Certifying Surgeon under the Factory and Workshop Act for the Newport District of the county of Salop. **Richard Hedden**, L.R.C.P.Lond., M.R.C.S., L.S.A., Medical Officer to the Union Workhouse by the Honiton (Devon) Board of Guardians. **Harold Kerr**, M.B., Ch.B.Edin., D.P.H.Camb., Assistant Medical Officer of Health to the County Borough of Bury. **J. H. Lumsden**, M.B., M.S.Aberd., Certifying Surgeon under the Factory and Workshop Act for the Denny District of the county of Stirling. **J. A. Pottinger**, M.D., Ch.B.Edin., Resident Surgeon to the Provincial Hospital, Port Elizabeth, South Africa. **George Frederick Rossiter**, M.B.Lond., M.R.C.S.Eng., Honorary Consulting Surgeon to the Weston-super-Mare Hospital. **J. Herbert Sanders**, M.D., M.R.C.S., L.R.C.P.Lond., Honorary Assistant Anæsthetist to the Central London Throat and Ear Hospital, W.C. **F. M. Sandwith**, M.D.Durh., F.R.C.P.Lond., Lecturer on Tropical Diseases at St. Thomas's Hospital Medical School. **Percy W. G. Sargent**, M.A., M.B., B.C.Cantab., F.R.C.S.Eng., Assistant Surgeon to St. Thomas's Hospital. **F. W. Schofield**, M.B., Ch.B.Vict., Second Assistant Medical Officer to the City Hospital, Sheffield. **Sydney Stephenson**, M.B., C.M.Edin., Ophthalmic Surgeon to the Queen's Jubilee Hospital. **William Henry Symons**, M.D.Brux., D.P.H.Oxon. and Durh., Superintendent to the Bath Statutory Hospital for Infectious Diseases at Combe Down. **George H. Temple**, M.B., C.M.Edin., Honorary Surgeon to the Weston-super-Mare Hospital. **John Brosnan Tighe**, M.B., B.Ch.Irel., Senior Assistant Medical Officer to the North Riding Asylum of Yorkshire. **Rupert Waterhouse**, M.D.Lond., L.R.C.P.Lond., Assistant Physician to the Royal United Hospital, Bath

### XMAS PRESENTS

### A GOOD NURSING BOOK

IS ALWAYS A MOST ACCEPTABLE

### Xmas Present to a Nurse.

### A SELECTION

***Complete Catalogue sent post free on application.***

**HOW TO BECOME A NURSE: How and Where to Train.**
Edited by Sir Henry Burdett, K.C.B.
2/- net, 2/4 post free.

**A HANDBOOK FOR NURSES.**
By J. K. Watson, M.D.
5/- net, 5/4 post free.

**NURSING: ITS THEORY AND PRACTICE.**
By Percy G. Lewis, M.D.
3/6 post free.

**NURSING: ITS PRINCIPLES AND PRACTICE.**
By Isabel Hampton.
7/6 net, 7/10 post free.

**THE NURSE'S PRONOUNCING DICTIONARY OF MEDICAL TERMS AND NURSING TREATMENT.**
By Honnor Morten.
New Edition by Mary I. Burdett.
Cloth, 2/- post free.
In handsome leather gilt, 2/6 net, 2/8 post free.

**MEDICAL GYMNASTICS, including SCHOTT (NAUHEIM) MOVEMENTS.**
By Axel V. Grafstrom, B.Sc., M.D.
2/6 post free.

**ELEMENTARY TREATISE ON THE LIGHT TREATMENT FOR NURSES.**
By James H. Sequeira, M.D. Lond.
2/6 net, 2/9 post free.

**PRACTICAL GUIDE TO SURGICAL BANDAGING AND DRESSINGS.**
By Wm. Johnson Smith, F.R.C.S.
2/- post free.

**NURSING HINTS TO PROBATIONERS ON PRACTICAL WORK.**
By Annesley Voysey.
2/- net, 2/4 post free.

**MEDICAL ELECTRICITY AND THE LIGHT TREATMENT.**
By Sister Kate Neale.
Illustrated. 2/6 net, 2/9 post free.

**ELEMENTARY PHYSIOLOGY FOR NURSES.**
By C. F. Marshall, M.D., B.Sc., F.R.C.S.
Crown 8vo., Illustrated. Cloth, 2/- post free.

**ELEMENTARY ANATOMY AND SURGERY FOR NURSES.**
By William McAdam Eccles, M.S. Lond., M.B., F.R.C.S., L.R.C.P.
2/6 post free.

**A COMPLETE HANDBOOK OF MIDWIFERY.**
By J. K. Watson, M.D. Edin.,
Author of "A Handbook for Nurses."
6/- net, 6/4 post free.

**HOSPITAL SISTERS AND THEIR DUTIES.**
By Eva C. Lückes, Matron, London Hospital.
2/6 post free.

**SURGICAL WARD-WORK AND NURSING.**
By Alexander Miles, M.D. Edin., C.M., F.R.C.S.E.
3/6 net, 3/10 post free.

**ART OF MASSAGE.**
By Mrs. A. Creighton Hale.
6/- post free.

**The Scientific Press, Ltd.**

**Publishers of Nursing Text-Books, Charts, and Case-Books of all Descriptions.**

**28 & 29 SOUTHAMPTON STREET, STRAND, LONDON, W.C.**

### Chatto & Windus's New Books.

**LIFE IN MOROCCO.** By Budgett Meakin, Author of 'The Land of the Moors.' With 24 Full-page Illustrations, demy 8vo. cloth, 12s. 6d. net.

**A HISTORY OF OUR OWN TIMES FROM 1897 to the Accession of King Edward VII.** By Justin McCarthy. 2 vols., demy 8vo. cloth, 24s. 'An admirable and impressive survey.'—*Standard.*

**PRAYERS WRITTEN AT VAILIMA.** By R. L. Stevenson. Post 8vo. half-cloth, 1s. net; leather, 2s. net. 'Will appeal to all his admirers, for they present the essence of the man.'—*Athenæum.*

**NEW SIX-SHILLING NOVELS.**

**THE WATERS OF DESTRUCTION.** By Alice Perrin, Author of 'East of Suez,' &c. 'A novel to be read. . . . Our interest never flags.'—*Times.*

**A THIEF IN THE NIGHT.** By E. W. Hornung, Author of 'Stingaree,' &c. 'Assuredly one of the best raffles in detective literature—all prizes of enjoyment, no blanks of insipidity.'—*Liverpool Post.*

**DILYS.** By F. E. Penny, Author of 'The Sanyasi.' 'Mrs. Penny has fairly made good her right to a front place in the still scanty roll of novelists of Anglo-India.'—*Guardian.*

**THE SPECULATIONS OF JOHN STEELE.** By Robert Barr. 'Has a breathless fascination about it.' *Evening Standard.*

**MAURICE.** By Joseph Keating, Author of 'Son of Judith.' 'One of the outstanding stories of the year.'—*Pall Mall Gazette.*

London: CHATTO & WINDUS, 111 St. Martin's Lane, W.C.

### CATHARINE GRACE LOCH,

ROYAL RED CROSS,
SENIOR LADY SUPERINTENDENT QUEEN ALEXANDRA'S MILITARY NURSING SERVICE FOR INDIA.

### A MEMOIR,

Edited by A. F. BRADSHAW, M.A. (Oxon.), C.B.,
SURGEON MAJOR-GENERAL,
HONORARY PHYSICIAN TO HIS MAJESTY THE KING.

WITH AN INTRODUCTION
By FIELD-MARSHAL THE EARL ROBERTS,
V.C., K.G., O.M.

Crown 8vo., cloth, with two portraits, 4s. net.

**COUNSELS AND IDEALS FROM THE WRITINGS OF** WILLIAM OSLER. Compiled by C. N. B. CAMAC. Crown 8vo., cloth, gilt top, with portrait and facsimile, 4s. net.

LONDON: HENRY FROWDE,
Oxford University Press Warehouse, Amen Corner, E.C.

### Rowntree's ELECT Cocoa

Owing to its exceptional richness in Theobromine, ROWNTREE'S ELECT COCOA is of the utmost value to Invalids and Convalescents, to whom its exceptional flavour is a further recommendation.

**Just the Book you want.** **The Duties of a Nurse Explained.**

It is essential that anyone desirous of taking up Nursing should have some idea of the duties of a Nurse.

### NURSING HINTS TO PROBATIONERS ON PRACTICAL WORK.

By ANNESLEY VOYSEY. Price 2/- net, 2/4 post free.

Contains full particulars of a Nurse's duties, and will prove of the greatest assistance to the Probationer.

*Of all Booksellers or of* THE SCIENTIFIC PRESS, Ltd., 28 & 29 Southampton Street, Strand, London, W.C.

